# Platelet-Rich Plasma in Reproductive Endocrinology: Mechanisms and Clinical Applications for Ovarian Reserve, PCOS, and Endometrial Receptivity

**DOI:** 10.3390/biomedicines13102488

**Published:** 2025-10-13

**Authors:** Zaher Merhi, Catrina Wiltshire McLeod, Fawziyah Shamim

**Affiliations:** 1 Department of Obstetrics and Gynecology, Division of Reproductive Endocrinology and Infertility, Albert Einstein College of Medicine, Bronx, NY 10461, USA; 2 Department of Obstetrics and Gynecology, Division of Reproductive Endocrinology and Infertility, Maimonides Medical Center, Brooklyn, NY 11219, USA; 3 Reproductive Endocrinology and Infertility, Rejuvenating Fertility Center, New York, NY 10019, USA; 4 Burrell College of Osteopathic Medicine, Melbourne, FL 32901, USA; 5 Department of Community Health and Health Sciences, CUNY Graduate School of Public Health and Health Policy, New York, NY 10027, USA

**Keywords:** platelet-rich plasma (PRP), ovarian rejuvenation, diminished ovarian reserve (DOR), premature ovarian insufficiency (POI), polycystic ovary syndrome (PCOS), endometrial receptivity, recurrent implantation failure (RIF), thin endometrium, infertility, assisted reproductive technology (ART)

## Abstract

Infertility remains a major global health concern, with diminished ovarian reserve (DOR), premature ovarian insufficiency (POI), polycystic ovary syndrome (PCOS), and impaired endometrial receptivity representing key contributors to poor assisted reproductive technology (ART) outcomes. Platelet-rich plasma (PRP), an autologous blood-derived concentrate enriched with growth factors and cytokines, has emerged as a promising regenerative therapy with angiogenic, anti-apoptotic, and proliferative properties. In reproductive medicine, intraovarian PRP has been evaluated for its potential to restore ovarian function in women with DOR and POI, improve oocyte competence and embryo euploidy, and promote ovulation in PCOS. Similarly, intrauterine PRP infusion or subendometrial zone injections has shown encouraging results in women with recurrent implantation failure and thin endometrium, enhancing endometrial thickness, receptivity, and implantation potential. Evidence from preclinical animal models and early clinical studies suggests multi-level mechanisms of action, including modulation of endocrine pathways, reduction in oxidative stress, activation of dormant follicles, and improvement of endometrial angiogenesis and receptivity. Despite these promising findings, results remain inconsistent due to heterogeneity in PRP preparation protocols, administration routes, timing, and study designs. Even though robust randomized controlled trials with standardized methodologies are needed to determine the efficacy and long-term reproductive outcomes of PRP in infertility treatment and anovulation in PCOS, PRP represents a novel and potentially transformative adjunct in reproductive endocrinology.

## 1. Introduction

Infertility affects an estimated 15% of couples worldwide, with female factors such as diminished ovarian reserve (DOR), polycystic ovary syndrome (PCOS), and impaired endometrial receptivity contributing significantly to reproductive challenges. These conditions are often associated with reduced oocyte yield, poor embryo quality, anovulation, and low implantation rates, significantly limiting the effectiveness of assisted reproductive technologies (ARTs). Conventional approaches—such as ovulation induction agents, gonadotropin stimulation, in vitro fertilization (IVF), oocyte donation, and endometrial priming—offer partial or symptomatic solutions but do not directly address the underlying pathophysiology. This unmet need has driven the exploration of novel therapies capable of improving ovulation as well as ovarian and endometrial function.

Platelet-rich plasma (PRP), derived from a patient’s own blood, an autologous concentrate enriched with growth factors including as Platelet-Derived Growth Factor (PDGF), Transforming Growth Factor-beta (TGF-β), Vascular Endothelial Growth Factor (VEGF), Insulin-like Growth Factor (IGF), among others [1], has emerged as a regenerative therapy in multiple fields of medicine and has been widely applied in orthopedics [2], dermatology [3], and wound healing [4] for its regenerative, angiogenic, and anti-apoptotic properties [5,6,7,8]. In reproductive medicine, PRP has been studied for its potential to restore ovarian function in women with DOR or premature ovarian insufficiency (POI) [8,9,10,11,12], to enhance endometrial receptivity in cases of thin endometrial lining (EMT) or recurrent implantation failure (RIF) [7], and more recently to improve ovulatory and hormonal profiles in PCOS [13,14]. The existing preclinical and early clinical studies have reported encouraging findings—including improvements in ovarian reserve markers (such as anti-Mullerian hormone [AMH]), antral follicle count (AFC), oocyte quality, blastocyst formation, and implantation rates—yet results remain inconsistent, and robust randomized controlled trials (RCTs) are scarce. Therefore, a critical synthesis of available evidence is warranted to evaluate the potential role of PRP across ovarian and endometrial domains.

## 2. Materials and Methods

A systematic literature search was performed across PubMed, Web of Science, and Scopus for articles published up to August 2025. The search strategy employed combinations of the following keywords: “platelet-rich plasma” OR “PRP” AND (“diminished ovarian reserve” OR “poor ovarian response” OR “premature ovarian insufficiency” OR “primary ovarian failure” OR “ovarian aging” OR “PCOS” OR “polycystic ovary syndrome” OR “endometrial receptivity” OR “thin endometrium” OR “recurrent implantation failure” OR “embryo development” OR “IVF” OR “assisted reproduction” OR “PCOS”). Filters were applied to include only original studies—randomized controlled trials, non-randomized trials, prospective or retrospective cohort studies, case series, case reports, and experimental animal studies—published in English. For studies pertaining to the ovaries, the search results yielded 161 articles and studies pertaining to the uterus led to 113 results. Only four articles pertaining to PRP and PCOS were found. Reviews, commentaries, and editorials as well as case reports (except for PCOS) were excluded unless they provided relevant mechanistic insights. Animal studies pertaining to the effect of PRP on ovarian function were also excluded. After exclusion criteria, the results 23 relevant publications.

Data extracted from eligible studies included: study design, sample size, patient or animal population, PRP preparation and activation method, platelet concentration, route and timing of administration, number and volume of injections, cycle context, primary outcomes (ovarian reserve markers, oocyte/embryo yield, endometrial thickness, implantation, pregnancy, live birth), adverse events, and follow-up duration. Outcomes were categorized into three domains—ovarian that included PCOS, endometrial, and embryonic—for structured synthesis.

## 3. Results

### 3.1. PRP Administration into the Ovaries

#### 3.1.1. Novel Application in Polycystic Ovary Syndrome (PCOS)

PCOS is a common endocrine disorder in reproductive-age women characterized by hyperandrogenism, chronic anovulation, hormonal imbalance, and polycystic ovarian morphology, and is closely associated with oxidative stress, insulin resistance, and obesity [15]. Disturbances in gonadotropin secretion, particularly elevated luteinizing hormone (LH), impaired estradiol (E2) and progesterone (P4) synthesis, and dysregulated expression of estrogen receptors (ERα and ERβ), contribute to impaired folliculogenesis, while overexpression of the pro-apoptotic factors further promotes granulosa cell death and follicular atresia [16,17,18,19]. Since PRP contains abundant growth factors such as PDGF, TGF-β, VEGF, IGF, among others [1] and has been used for its regenerative and anti-apoptotic potential in other tissues [20], several new studies explored its efficacy in PCOS.

The first evidence suggesting a role for PRP in PCOS emerged from a case report describing a 35-year-old woman with long-standing PCOS, obesity, type 2 diabetes, hypertension, hyperandrogenism, and more than one year of amenorrhea [21]. Baseline evaluation showed polycystic ovaries with >15 antral follicles each, markedly elevated testosterone (140.3 ng/dL), 17-hydroxyprogesterone (218 ng/dL), hyperglycemia, and elevated C-reactive protein (CRP; 44.4 mg/L). She underwent intraovarian PRP administration which was injected bilaterally into the ovarian cortex under ultrasound guidance (2 mL per ovary, 5–7 punctures). Within 10 days, a dominant follicle developed with appropriate E2 rise, ovulation was confirmed by luteal P4 and corpus luteum formation, and menses resumed. Hormonal improvements were also observed, including a reduction in testosterone from 140.3 to 20.8 ng/dL and a decline in CRP. During the subsequent cycle, follicular development and endocrine responses normalized, allowing an intrauterine insemination attempt, though conception did not occur. Notably, spontaneous ovulation and normalization of androgen levels persisted for up to three months. Although the possibility of a coincidental ovulatory event or an effect of ovarian puncture (similar to micro-drilling) cannot be excluded, the endocrine and follicular changes suggest a biologic effect of PRP. This case highlights the potential of intraovarian PRP to restore ovulation and improve endocrine balance in PCOS, while emphasizing the need for larger, controlled studies to confirm efficacy and assess long-term reproductive and metabolic outcomes.

Poor oocyte quality is a well-recognized contributor to suboptimal ART outcomes in women with PCOS [22,23]. Another case report described a 34-year-old woman with PCOS struggling with DOR (AFC = 0 in the right ovary and AFC = 2–3 in the left ovary, follicle-stimulating hormone (FSH) (10.99 IU/mL), who, along with her partner with severe teratozoospermia, presented with four years of primary infertility [13]. Previous treatment attempts, including two intrauterine insemination (IUI) cycles and one IVF, had failed, with only immature and degenerated oocytes retrieved. Following six months of lifestyle optimization and inositol therapy, she underwent intraovarian PRP into the ovaries under ultrasound guidance. After PRP, her AFC increased to 12, and ovarian stimulation resulted in the retrieval of seven oocytes (five mature MII, two MI), which produced multiple embryos. Although the initial fresh embryo transfer failed, a subsequent frozen embryo transfer led to a clinical pregnancy. This clinical improvement mirrors findings from other studies demonstrating that PRP may enhance ovarian reserve markers, oocyte yield, and pregnancy outcomes in poor ovarian responders with PCOS, potentially through angiogenic and follicle-activating effects of platelet-derived growth factors [24,25].

Seyyed Anvari et al. [14] conducted an experimental study to investigate whether PRP could reverse PCOS-related ovarian dysfunction in a rat model. Thirty immature Sprague-Dawley female rats were divided into five groups: untreated controls, PCOS induced for 15 or 30 days, and PCOS induced with subsequent PRP treatment for 15 or 30 days. PCOS was established by daily subcutaneous DHEA injections for 15 days, after which autologous PRP—activated with calcium chloride—was administered into the mesovarium surrounding the ovaries. The investigators assessed a broad range of outcomes, including serum sex hormones, oxidative stress markers, follicle counts and corpora lutea formation, as well as molecular and histological indicators of ovarian health. Compared with untreated PCOS animals, those receiving PRP exhibited preservation of preantral and antral follicles, new corpora lutea consistent with ovulation, and reduced RNA damage. PRP also enhanced Er-α and Er-β expression, downregulated c-Myc, and shifted the balance away from apoptosis toward follicular development. Oxidative stress was mitigated, with increases in TAC, SOD, and GSH-px alongside reductions in MDA. Endocrine function improved as well, with normalization of gonadotropins and androgens and restoration of E2 and P4 production. Collectively, these findings suggest that PRP exerts multi-level benefits in PCOS, combining endocrine regulation, antioxidant reinforcement, anti-apoptotic signaling, and folliculogenic support.

Conventional pharmacological options for PCOS, such as metformin and clomiphene, are often hampered by limited efficacy and side effects [26,27], underscoring the need for novel regenerative strategies that can simultaneously address ovarian and metabolic dysfunction. Placenta-derived mesenchymal stem cells (PDMSCs) possess well-documented immunomodulatory, anti-inflammatory, angiogenic, and regenerative properties [28], while PRP has also shown favorable effects on ovarian function [29,30]. To explore their potential individually and in combination, a recent study [31] employed a letrozole-induced rat model of PCOS. Twenty-five adult Wistar rats were randomized into sham, PCOS, PDMSCs, PRP, and PDMSCs + PRP groups (*n* = 5 each). PDMSCs were derived from term human placentas (CD73^+^, CD90^+^, CD105^+^, CD34^−^, CD45^−^), whereas PRP was isolated from rat blood and activated with thrombin and calcium chloride; both treatments were administered as single intraovarian injections, with outcomes assessed 14 days later. Compared with untreated PCOS rats—which displayed cystic follicle formation, impaired folliculogenesis, absent corpora lutea, hyperandrogenism, gonadotropin imbalance, hyperglycemia, insulin resistance, and elevated pro-inflammatory cytokines—the PDMSCs and PRP groups each demonstrated partial restoration of ovarian and metabolic parameters. Notably, the combination therapy PDMSCs + PRP exerted synergistic effects, improving follicular development across all stages, restoring corpora lutea, lowering testosterone and LH, normalizing FSH and E2, reducing insulin resistance indices, and markedly suppressing TNF-α and IL-6. Mechanistic insights pointed to anti-apoptotic, angiogenic, and paracrine effects involving PI3K/AKT and TGF-β signaling, enhanced granulosa cell survival, modulation of steroidogenesis, improved GLUT4-mediated glucose uptake, and attenuation of NF-κB–driven inflammation.

**Figure 1:**
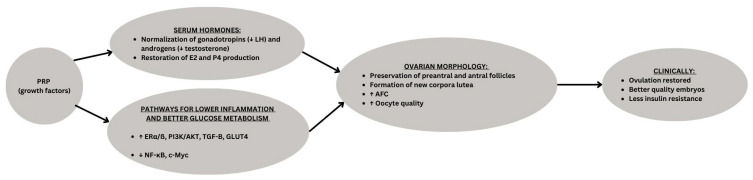
Conceptual schematic of Platelet-Rich Plasma (PRP) effects in PCOS. PRP enriched with growth factors, acts on the ovary by restoring hormonal balance and modulating molecular pathways. These changes promote folliculogenesis, corpus luteum formation, and improved oocyte quality, ultimately leading to clinical benefits such as resumption of ovulation, improved embryo development, and pregnancy. ↑ means “increase in”, ↓means “decrease in”, underline is the category being presented.

Although limited by small sample size, short duration, and reliance on an animal model without fertility endpoints, or case reports in humans, these studies suggest that PRP represents a promising regenerative approach for PCOS by targeting reproductive, metabolic, and inflammatory pathways simultaneously (Figure 1). Further translational and clinical research is warranted to validate their therapeutic potential in humans.

#### 3.1.2. Ovarian Aging/Insufficiency

Ovarian aging leading to DOR and POI are significant causes of female infertility characterized by reduced quantity and/or quality of oocytes [32]. According to recent data, the incidence of POI has significantly increased from 1% to 3.5% [32]. Thus, finding solutions to help this patient population conceive using their own DNA remains a challenge. One of the potential adjunct to improving fertility is intraovarian PRP administration that was first reported by a Greek team that reported efficacy by leading to a pregnancy and live birth even in menopausal women [33,34]. Small pilot studies and case reports began to emerge around 2018, showing promising results such as improved hormone levels, increased AFC, higher oocyte yield, and even spontaneous pregnancies [35]. These preliminary findings led to the introduction of intraovarian PRP injections as an experimental intervention, which is now increasingly utilized in clinical practice, particularly as an adjunct to IVF for women who, for ethical or religious reasons, cannot pursue donor oocytes. In this section, we will focus on human studies.

##### Human Studies: From Pilot Studies to RCT (Table 1)

In one of the first pilot studies, four women with DOR and a history of poor IVF response underwent intraovarian injection of autologous PRP activated with calcium gluconate [35]. Patients were followed with serum AMH, FSH, and E2 measurements at two-week intervals, and IVF was performed when ovarian reserve markers showed improvement. Results demonstrated a significant reduction in FSH (from 13.6 to 7.7 mIU/mL, *p* < 0.01) and a non-significant increase in AMH (0.38 to 0.61 ng/mL, *p* = 0.17). Oocyte retrieval yielded 4–7 mature oocytes per patient, with all subjects producing at least one blastocyst suitable for cryopreservation, and one case resulting in a clinical pregnancy.

In a before-and-after clinical study from Iran [10], 22 infertile women with DOR, defined by the Bologna criteria, underwent intraovarian infusion of autologous PRP following oocyte retrieval during IVF cycles. Baseline AMH levels and AFC were measured prior to the procedure and re-evaluated three months later. Results showed a significant increase in AMH after PRP (0.24 ± 0.20 vs. 0.99 ± 0.80 ng/mL, *p* < 0.001), while AFC showed a non-significant upward trend (4.71 ± 2.23 vs. 5.66 ± 2.63, *p* = 0.14). Logistic regression analysis revealed that age and BMI did not influence ovarian response, though infertility duration >5 years was associated with a greater likelihood of persistently low AFC.

In a recent prospective case–control study conducted in Taiwan, IVF patients (*n* = 74) with a history of at least two failed cycles without good-quality blastocyst formation were evaluated for the effect of intraovarian PRP injection [36]. Forty-four patients elected to receive autologous PRP that was injected into both ovarian cortices at four sites during the follicular phase under transvaginal ultrasound guidance, while 30 patients served as controls. All participants underwent two controlled ovarian hyperstimulation (COH) cycles with PGT-A; the PRP group had one cycle before and one after PRP administration. In the second COH cycle, the PRP group showed significantly higher numbers of fertilized oocytes (5.2 ± 3.6 vs. 3.3 ± 3.5, *p* = 0.011), total blastocysts (1.7 ± 1.5 vs. 0.5 ± 0.7, *p* < 0.0001), and good-quality blastocysts (0.6 ± 0.8 vs. 0.0 ± 0.2, *p* < 0.0001) compared with controls, as well as superior total (35% vs. 13%) and good-quality blastocyst rates (14% vs. 1%). Benefits were most pronounced when COH was performed one to two months post-PRP but diminished after three months. Among PRP patients, 22 produced blastocysts suitable for PGT-A, with an euploidy rate of 17.6% and a 29% clinical pregnancy rate after transfer of euploid or mosaic embryos. The authors concluded that intraovarian PRP may improve blastocyst yield and quality in women with recurrent IVF failure.

Merhi et al. [24] investigated the effect of intraovarian PRP on embryo genetics in infertile women with previous failed IVF cycles. Twelve patients underwent two IVF cycles using identical mild stimulation protocols: cycle 1 served as baseline, after which each woman received autologous intraovarian PRP. Within three months, all participants underwent a second IVF cycle with PGT-A. Clinical parameters—including FSH, AFC, oocytes retrieved, and number of good-quality blastocysts—did not differ significantly between cycles. However, euploidy rates improved markedly: only 3 of 37 embryos (8.1%) were euploid pre-PRP, compared with 11 of 28 embryos (39.3%) post-PRP (*p* = 0.002), and three clinical pregnancies were achieved. The authors concluded that intraovarian PRP may enhance oocyte competence and improve embryo euploidy, possibly through growth factor-mediated effects on meiotic regulation.

In a prospective cohort study from Greece, Potiris et al. [37] evaluated the effect of intraovarian PRP infusion in 32 women ≥40 years with infertility due to anovulatory cycles. PRP was prepared from 65 to 70 mL autologous blood via double centrifugation (target ~1,000,000 platelets/µL) and injected bilaterally into the ovarian cortex under transvaginal ultrasound guidance using a 17-gauge needle. Each participant received two courses of PRP treatment during the early follicular phase over four months. Hormonal profiles (FSH, LH, estradiol (E2), progesterone (P4), prolactin, testosterone, cortisol), AFC, and metabolic markers (vitamin D, cholesterol, triglycerides, liver/kidney function tests) were assessed at baseline, between injections, and up to two months post-treatment. Results demonstrated a 75% increase in AFC and significant reductions in FSH (17.9 → 8.4 mIU/mL) and LH (15.1 → 6.9 mIU/mL), along with decreased prolactin and improved metabolic parameters, including higher vitamin D and lower cholesterol/triglycerides. While ovarian reserve and hormonal balance improved, clinical reproductive outcomes (ovulation, conception, live births) were not yet reported. The authors concluded that intraovarian PRP may restore follicular activity and improve endocrine function in anovulatory women of advanced age.

In a large retrospective multicenter cohort study, Molinaro et al. [12] evaluated the impact of bilateral intraovarian PRP injections on ovarian reserve and IVF outcomes in 353 women ≤45 years old with either DOR (*n* = 207) or POI (*n* = 146) who declined oocyte donation. PRP was prepared from autologous blood, activated with calcium chloride, and injected under ultrasound guidance; outcomes included AFC, AMH, ovarian stimulation (OS) responses, IVF results, and reproductive outcomes. In DOR patients, PRP significantly increased AFC at all follow-up visits (baseline 2.6 vs. peak 5.3, *p* < 0.0001), while AMH rose only transiently at first follow-up. Although the number of oocytes retrieved and blastocysts obtained were unchanged, oocyte maturation (65.8% → 80.8%, *p* = 0.003), fertilization (61.6% → 75.8%, *p* = 0.011), and cleavage rates (61.6% → 73.9%, *p* = 0.03) improved, with trends toward higher implantation (9.4% → 35.1%), biochemical pregnancy (12.5% → 41.5%), and live birth rates (0% → 17.6%). Twenty-three clinical pregnancies and seven live births were reported among DOR patients, including six natural conceptions. In contrast, POI patients showed only modest AFC gains (1.0 → 2.1, *p* < 0.0001) without improvement in AMH, IVF yield, or reproductive outcomes, though six pregnancies and one live birth occurred. The authors concluded that PRP did not enhance quantitative ovarian output but improved oocyte quality parameters in DOR, while efficacy in POI was minimal.

In the first double-blind, placebo-controlled randomized trial of intraovarian PRP, Barrenetxea et al. [9] investigated its effect on IVF outcomes in 60 women (30–42 years) with DOR according to POSEIDON groups 3 and 4. Patients underwent three consecutive stimulation cycles: during the first oocyte retrieval, the treatment group received bilateral intraovarian injections of autologous PRP (4 mL per ovary, prepared from 15 mL peripheral blood, activated with CaCl_2_), while controls received sham saline injections. In subsequent cycles, mature oocyte yield, blastocyst development, euploidy, and pregnancy outcomes were compared. Cumulatively, PRP patients had a modest but statistically significant increase in mature oocytes (10.45 ± 0.41 vs. 8.91 ± 0.39; *p* = 0.008), with the greatest difference noted at the third retrieval (5.27 vs. 4.15; *p* = 0.029). However, no differences were observed in blastocyst development, euploidy rates (both 0.81 euploid blastocysts per patient), or live births. Surprisingly, clinical pregnancy rates were higher in controls (60%) compared with the PRP group (27%, *p* = 0.018). The authors concluded that while PRP may modestly increase the number of mature oocytes retrieved, it did not improve embryo genetics or clinical outcomes, suggesting possible non-specific or mechanical effects rather than true ovarian rejuvenation.

##### How Many Intraovarian PRP Injections Are Needed?

A study aimed to evaluate the effects of one-time versus two-time PRP injections on ovarian reserve and IVF outcomes in DOR patients, providing novel insights into optimizing PRP protocols [11]. In that study [11], 71 women with poor ovarian response (DOR; POSEIDON groups 3 or 4, AMH < 1.2 ng/mL, AFC < 5) underwent autologous intraovarian PRP injections to assess whether single versus double treatments offered differential benefits. Patients subsequently underwent IVF/ICSI, and pre-PRP and post-PRP treatment ovarian reserve markers and cycle outcomes were compared. Overall, PRP significantly increased AMH (0.33 → 0.43 ng/mL, *p* = 0.005) and AFC (2.62 → 3.80, *p* < 0.001), as well as peak E2, number of large follicles ≥14 mm, retrieved oocytes (2.32 → 3.59, *p* < 0.001), normally fertilized zygotes, and high-quality cleavage embryos. Subgroup analysis revealed that both single and double PRP injections improved ovarian reserve and IVF outcomes, but no significant differences were found between one versus two treatments, although AMH increase reached significance only in the double-injection group. The authors concluded that a single PRP injection may be as effective as two, offering a simpler and more cost-effective protocol for DOR patients.

Aflatoonian et al. [38] conducted one of the few studies to explore a repeat-dosing strategy for intraovarian PRP. In this before–after trial, 17 poor ovarian responders (PORs) and 9 women with primary ovarian insufficiency (POI) received intraovarian PRP (1.5 mL per ovary), with most participants undergoing a second injection at a higher volume (3 mL per ovary) three months later. The rationale for this two-step protocol was to potentially enhance and sustain ovarian recovery in severely depleted ovaries, in contrast to most other studies that used only a single PRP administration. Results showed no significant improvement in AMH, FSH, LH, or estradiol levels; however, nearly half of the POR group conceived spontaneously, resulting in four live births, three miscarriages, and one ongoing pregnancy. In the POI group, 22% experienced menstrual restoration after the second injection, but no pregnancies occurred. The authors concluded that PRP may offer some benefit in poor responders but is of limited value in POI. Key limitations included small sample size, lack of a control arm, and the absence of measurable hormonal benefit despite spontaneous pregnancies, suggesting that repeating PRP at a higher dose may not provide additional efficacy compared to single-injection protocols.

A key limitation of the current body of evidence is the lack of consensus regarding the optimal frequency of intraovarian PRP administration. While some studies report benefits after a single injection, others have explored repeated treatments; however, no standardized protocol exists. As such, it remains unclear how many PRP sessions are required to achieve the best outcomes in terms of oocyte quality and ovarian response. Well-designed, prospective trials are needed to establish evidence-based guidelines in this area.

### 3.2. PRP Administration into the Uterus: Infusions Versus Injections (Table 2)

#### 3.2.1. Intrauterine PRP Infusion

**Figure 2:**
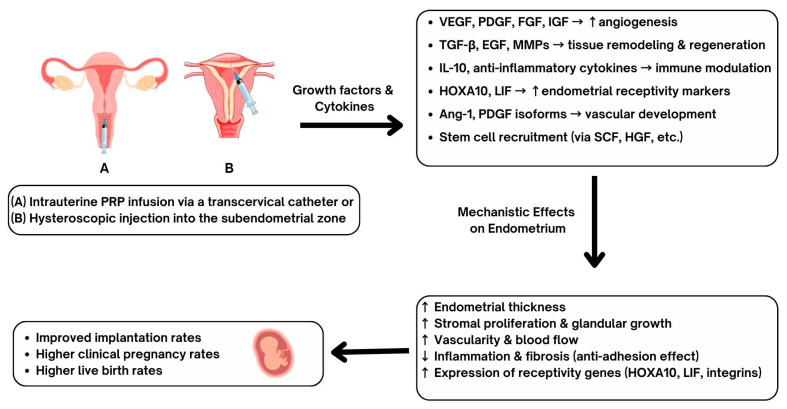
Proposed mechanisms and clinical outcomes of intrauterine platelet-rich plasma (PRP) administration. PRP can be administered into the endometrium via transcervical catheter (**A**) or by hysteroscopic injections (**B**), allowing for administration of growth factors and cytokines including VEGF (vascular endothelial growth factor), PDGF (platelet-derived growth factor), FGF (fibroblast growth factor), IGF (insulin-like growth factor), TGF-β (transforming growth factor-β), EGF (epidermal growth factor), MMPs (matrix metalloproteinases), IL-10 (interleukin-10), HOXA10 (homeobox A10), LIF (leukemia inhibitory factor), Ang-1 (angiopoietin-1), SCF (stem cell factor), and HGF (hepatocyte growth factor). These biological mediators promote angiogenesis, tissue remodeling, immune modulation, vascular development, and stem cell recruitment. Mechanistic effects on the endometrium include increased endometrial thickness, stromal proliferation, glandular growth, vascularity, and receptivity gene expression. Reduced endometrial inflammation and fibrosis have been demonstrated to be part of the PRP benefits. Clinical outcomes include improved rates of embryo implantation, higher clinical pregnancy rates, and increased live birth rates have been reported following PRP administration.

In recent years, clinical studies have investigated the application of intrauterine PRP to improve EMT, implantation rates, and clinical pregnancy outcomes. Contemporary studies conducted in human subjects provide valuable insight about PRP’s clinical utility, particularly among patients with RIF and thin endometrium (Figure 2). In a randomized controlled trial, Zamaniyan et al. [39] assessed whether intrauterine PRP could improve pregnancy outcomes in women with RIF. A total of 120 infertile women aged 20–40 years, with ≥3 failed transfers of good-quality embryos, were randomized to receive either standard frozen-thawed embryo transfer (control, *n* = 60) or intrauterine infusion of 0.5 mL autologous PRP (platelet concentration 4–7× baseline, prepared by double centrifugation) administered 48 h before transfer (intervention, *n* = 60). All patients underwent standard endometrial preparation with E2 valerate and P4 support, and blastocyst transfer was performed on day 5 of P4 administration. Of the 98 women who completed the study, baseline demographics and IVF cycle characteristics were similar between groups, though EMT on hCG day was greater in the PRP arm. Clinical outcomes were significantly improved with PRP: clinical pregnancy rates (48.3% vs. 23.3%, *p* = 0.001), ongoing pregnancy rates (46.7% vs. 11.7%, *p* = 0.001), and implantation rates (58.3% vs. 25%, *p* = 0.001) were all higher in the intervention group. Miscarriage and multiple pregnancy rates did not differ significantly. The authors concluded that intrauterine PRP prior to embryo transfer enhances endometrial receptivity and improves IVF outcomes in RIF patients.

In an unblinded randomized clinical trial conducted between 2020 and 2022, Mehrafza et al. [5] compared intrauterine infusion of PRP with granulocyte colony-stimulating factor (G-CSF) in 200 women < 41 years old with RIF (≥2 failed transfers of high-quality embryos). Participants were randomized to receive either 1 mL autologous PRP (prepared from 8.5 mL venous blood via double centrifugation to yield a 4–5× platelet concentration) infused 48 h before embryo transfer, or 1 mL intrauterine G-CSF (300 µg, Filgrastim 30 mIU/mL) administered on the first day of P4 supplementation. All patients underwent standardized FET cycles with E2 valerate for endometrial priming and vaginal P4 for luteal support. Outcomes included implantation, chemical pregnancy, clinical pregnancy (ultrasound-detected sac with heartbeat), and ongoing pregnancy ≥ 12 weeks. Their results showed significantly higher implantation rates (*p* = 0.014), chemical pregnancy (36.7% vs. 17.4%, *p* = 0.003), clinical pregnancy (33.7% vs. 13%, *p* = 0.001), and ongoing pregnancy (27.6% vs. 13%, *p* = 0.020) in the PRP group compared to G-CSF. The authors concluded that intrauterine PRP is more effective than G-CSF in enhancing endometrial receptivity and pregnancy outcomes in RIF patients.

In a prospective single-arm, self-controlled trial conducted in Japan, Kusumi et al. [40] investigated the efficacy of intrauterine PRP infusion in women with thin EMT (≤7 mm) and RIF undergoing FET with HRT. Thirty-nine patients were enrolled, of whom 36 received intrauterine PRP (1 mL prepared from 20 mL autologous blood via centrifugation) administered twice on cycle days 10 and 12 during the second HRT cycle. The EMT was assessed by blinded and unblinded ultrasound measurements, and FET was performed thereafter. PRP significantly increased EMT: mean gains from baseline to cycle day 14 were 1.27 mm (unblinded, *p* < 0.001) and 0.72 mm (blinded, *p* = 0.001). Of the 36 patients, 32 underwent FET, yielding an implantation rate of 13.9%, chemical pregnancy rate of 18.8%, and clinical pregnancy rate of 15.6%, with three live births ultimately reported. No adverse events occurred. The authors concluded that intrauterine PRP is a safe and effective strategy to improve EMT and potentially enhance implantation in patients with refractory thin EMT. In a retrospective cohort study from Turkey, Gürkan and Alper [41] evaluated the effect of intrauterine PRP infusion on fertility outcomes in 150 infertile women with either RIF, thin EMT, or both, undergoing FET cycles. Autologous PRP was infused into the endometrial cavity via IUI catheter on day 10 of E2 replacement; embryo transfer was performed on day 5 once EMT exceeded 7 mm. Outcomes were compared with 150 age-matched controls with normal endometrium and unexplained infertility, and a subgroup of 96 RIF patients without PRP. PRP significantly increased EMT overall (7.38 → 7.96 mm, *p* < 0.001), with notable improvement in women with thin EMT (5.85 → 6.65 mm, *p* < 0.001). However, clinical pregnancy and live birth rates were not significantly higher with PRP: in RIF patients, rates were similar between PRP and no-PRP groups (34.3% vs. 34.4%), and overall pregnancy outcomes did not differ significantly between PRP-treated patients and controls. The authors concluded that while intrauterine PRP effectively increase EMT, it does not significantly improve implantation or live birth rates.

In a prospective cohort study from Korea, Shin et al. [42] evaluated the efficacy and mechanistic basis of intrauterine PRP infusion in 91 women with refractory thin endometrium and ≥2 failed IVF cycles. Patients had undergone at least two prior therapies for thin lining without success and received autologous PRP prepared from 18 mL venous blood (platelet concentration 717–1565 × 10^3^/µL), activated with calcium gluconate, and infused into the uterine cavity every three days (maximum three infusions) during hormone replacement FET cycles until EMT reached 7 mm. Compared with the previous cycle without PRP, the PRP cycle showed significant improvements in total pregnancy rate (14.3% → 42.9%), implantation rate (3.1% → 16.8%), clinical pregnancy rate (3.3% → 31.9%), and live birth rate (0% → 20.9%) (all *p* < 0.001), alongside a mean EMT gain of 0.8 mm. Cytokine assays demonstrated PRP enrichment in pro-angiogenic mediators, including Ang-1, EGF, LAP (TGF-β1), MMP-8, and PDGF isoforms, supporting angiogenesis and tissue remodeling as underlying mechanisms. Obstetric follow-up revealed 19 live births, though placenta accreta spectrum disorders occurred in 21% of cases. The authors concluded that PRP improves EMT and reproductive outcomes in this difficult cohort, likely through angiogenic signaling. Limitations included its single-arm design without a control group, modest EMT gains (remaining <7 mm on average), heterogeneity in endometrial etiology, and increased risk of abnormal placentation in women with prior uterine trauma.

In a retrospective cohort study from Canada, Russell et al. [43] evaluated intrauterine PRP infusion in 85 women with RIF, thin EMT, or both, undergoing frozen embryo transfer with PGT-A-tested euploid embryos. PRP was infused into the uterine cavity between cycle days 10–15, with repeat doses if EMT remained <7 mm. Across 133 cycles and 211 infusions, PRP significantly increased median EMT from 6.7 to 7.6 mm (*p* < 0.0001), with most patients responding after a single infusion. Compared to patients’ prior non-PRP cycles, biochemical pregnancy rates improved (48.3% vs. 35.5%), as did clinical pregnancy (37.1% vs. 20.2%) and live birth rates (19.6% vs. 2.9%), while miscarriage rates decreased. Improvements were most consistent among RIF patients, though benefits were also seen in this EMT cases. The authors concluded that intrauterine PRP enhances EMT and improves implantation and live birth in difficult infertility populations.

In a 2024 meta-analysis, Liu et al. [44] systematically reviewed randomized controlled trials (RCTs) to assess the efficacy of intrauterine PRP infusion in women with thin EMT undergoing ART. A comprehensive search of PubMed, Embase, Cochrane Library, Web of Science, and MEDLINE through June 2024 identified eight RCTs involving 678 patients (333 PRP vs. 345 controls). PRP infusion was repeated if EMT remained <7 mm), and outcomes were compared with HRT or placebo. Pooled analysis showed that PRP significantly improved EMT (increase by 1.23 mm, 95% CI 0.87–1.59), clinical pregnancy (RR 2.04, 95% CI 1.52–2.76), live birth (RR 2.46, 95% CI 1.57–3.85), implantation (RR 2.71, 95% CI 1.91–3.84), and reduced cycle cancelation rates (RR 0.46, 95% CI 0.23–0.93). No significant effects were observed for chemical pregnancy, spontaneous abortion, or vascular improvement.

**Table 2:** Summary of Human Studies on Intrauterine Infusion and Hysteroscopic Injection of PRP.

Author/Year	Study Design/Population	PRP Method	Outcomes	Conclusions
Zamaniyan et al. (2021) [39]	RCT, 120 women with RIF	0.5 mL intrauterine PRP infusion before ET	↑ EMT, ↑ implantation, ↑ clinical pregnancy, ↑ ongoing pregnancy	PRP improved outcomes in RIF patients
Mehrafza et al. (2024) [5]	RCT, 200 women with RIF	1 mL intrauterine PRP vs. G-CSF	↑ implantation, ↑ chemical and clinical pregnancy, ↑ ongoing pregnancy (vs G-CSF)	PRP superior to G-CSF
Kusumi et al. (2020) [40]	Single-arm, 39 women with thin EMT + RIF	1 mL intrauterine PRP twice (CD10 and CD12)	↑ EMT (mean +0.7–1.3 mm), modest pregnancy rates (15.6% CPR), 3 live births	PRP safe, improved EMT, some pregnancies
Gürkan and Alper (2025) [41]	Retrospective, 150 infertile women (RIF/thin EMT)	Intrauterine PRP on CD10 of E2 cycle	↑ EMT, but no significant ↑ in pregnancy or live birth	PRP improved EMT but not outcomes
Shin et al. (2024) [42]	Prospective, 91 women with thin EMT, ≥2 failed IVF	Repeated intrauterine PRP infusions until EMT ≥7 mm	↑ EMT, ↑ pregnancy, ↑ live birth (20.9%), but 21% placenta accreta spectrum	PRP effective but safety concerns noted
Russell et al. (2022) [43]	Retrospective, 85 women with RIF/thin EMT	Intrauterine PRP during FET cycles	↑ EMT, ↑ biochemical/clinical pregnancy, ↑ live birth, ↓ miscarriage	PRP beneficial, especially for RIF
Efendieva et al. (2023) [45]	Pilot RCT, 115 infertile women with thin EMT	Hysteroscopic PRP ± autologous endometrial cells	↑ EMT, ↑ vascularization, some pregnancies (3 full-term deliveries with cell + PRP)	PRP injections improved EMT; cell + PRP promising
Agarwal et al. (2020) [46]	Pilot, 32 women with thin EMT, recurrent ET cancelations	Hysteroscopic subendometrial PRP (4 sites)	75% achieved EMT ≥ 7 mm, 42% CPR, 21% live birth	Hysteroscopic PRP effective in refractory EMT
Yu et al. (2024) [47]	Case–control, 116 women with thin EMT	PRP infusion vs. hysteroscopic injection vs. controls	Both PRP methods ↑ EMT; hysteroscopic PRP ↑ implantation (52%) and live birth (38%) > infusion	Hysteroscopic PRP more effective than infusion

**Abbreviations:** RIF: Recurrent Implantation Failure; ET: Embryo Transfer; PRP: Platelet-Rich Plasma; EMT: Endometrial Thickness; RCT: Randomized Controlled Trial; G-CSF: Granulocyte Colony-Stimulating Factor; CPR: Clinical Pregnancy Rate; FET: Frozen Embryo Transfer; CD: Cycle Day. ↑ means “increase in”, ↓ means “decrease in”.

#### 3.2.2. Hysteroscopic PRP Injections

In a pilot randomized study from Russia, Efendieva et al. [45] evaluated the efficacy of hysteroscopically guided intraendometrial PRP injections, with or without autologous endometrial cells, in 115 infertile women with refractory thin EMT (<7 mm at implantation window). Patients were randomized into four groups: Group 1 received conservative therapy (physiotherapy), Group 2 received a single intraendometrial PRP injection (injected 2–3 mm deep under hysteroscopic guidance), Group 3 received combined conservative therapy followed by PRP, and Group 4 (*n* = 5) received PRP reinforced with minimally manipulated endometrial cells obtained from biopsy. EMT increased significantly in all treatment groups, with the greatest gain observed in Groups 3 and 4, and Doppler ultrasound confirmed improved uterine microcirculation after PRP. Histological analysis demonstrated enhanced vascularization and cell proliferation, supported by elevated CD34 expression in women who achieved pregnancy. Clinically, implantation and live birth rates were numerically higher in PRP-treated groups compared to controls, with three full-term deliveries in the cell-PRP group, though differences were not statistically significant due to small sample sizes. The authors concluded that hysteroscopic intraendometrial PRP injections, especially when combined with autologous endometrial cells, improve EMT, receptivity, and microvascular function in women with refractory thin EMT.

In a prospective cross-sectional pilot study, Agarwal et al. [46] evaluated hysteroscopic instillation of PRP into the endomyometrial junction in 32 infertile women (aged 27–39 years) with recurrent embryo transfer cancelations due to refractory thin EMT (<7 mm). PRP was prepared and injected under hysteroscopic guidance into four quadrants of the subendometrial zone (1 mL per quadrant). Endometrial preparation involved OCP pretreatment, GnRH agonist down-regulation, and E2 supplementation, with luteal support provided by vaginal P4. Outcomes assessed included EMT, vascularity (Doppler), embryo transfer feasibility, and pregnancy rates. Following PRP, 75% of patients (24/32) achieved EMT ≥ 7 mm, with significant improvements in subendometrial blood flow; mean EMT gain was 1.5–2 mm. Of the 24 who underwent frozen embryo transfer, 12 (50%) conceived, including 10 clinical pregnancies (41.7%) and 5 live births (20.8%). Two patients experienced first-trimester miscarriage. The authors concluded that hysteroscopic PRP administration is a safe, well-tolerated, and potentially more effective method than intrauterine infusion for enhancing endometrial regeneration in thin-lining patients.

One study compared intrauterine infusion versus hysteroscopic injection of autologous PRP in 116 infertile women with persistent thin EMT (<7 mm) undergoing euploid FET cycles [47]. All participants had at least one prior failed euploid FET. Fifty-five women underwent intrauterine infusion (2 mL twice, 48 h apart), 38 received hysteroscopic subendometrial injection (2 mL divided into four quadrants, under anesthesia), and 23 women served as thin EMT controls on HRT alone; 30 women with normal lining (>7 mm) formed a reference group. Primary outcomes were EMT, implantation rate, clinical pregnancy rate, and live birth rate. Post-treatment, EMT exceeded 7 mm in 78.2% of the infusion group and 55.3% of the injection group. On transfer day, EMT was significantly thicker in both PRP groups than in controls (8.8 vs. 8.7 vs. 6.7 mm, *p* < 0.001). Implantation and live birth rate were highest with hysteroscopic PRP (52% implantation rate, 38% live birth rate), significantly outperforming controls (18% implantation rate, 4% live birth rate), whereas intrauterine infusion showed modest, non-significant improvements (27% implantation rate, 23% live birth rate). The authors concluded that PRP enhances EMT and pregnancy outcomes in thin endometrium, with hysteroscopic delivery showing greater impact on implantation and live birth rate.

## 4. Discussion

The accumulated evidence to date suggests that PRP holds promise as a regenerative therapy in reproductive endocrinology. In women with DOR or POI, intraovarian PRP has been associated with improvements in surrogate markers of ovarian reserve (AMH, AFC), oocyte maturation, fertilization, and embryo development, with some studies reporting spontaneous conceptions or pregnancies naturally or following IVF. Notably, one study demonstrated a significant increase in embryo euploidy rates after PRP, suggesting potential benefits at the genomic level. Although the multicenter study by Molinaro et al. [12] adds to the discussion on intraovarian PRP, its retrospective design, methodological choices, and outcome reporting raise significant concerns. A major issue is the PRP administration procedure. The authors reported injecting ~2 mL of PRP per ovary in 2–3 aliquots, targeting both cortical and stromal regions. It appears that patients receiving two injections per ovary had one delivered subcortically and one in the stroma. This approach is problematic for two reasons: first, the total volume (2 mL) is approximately 50% lower than that used in most prior studies [9]; and second, allocating half the volume to the stromal compartment is questionable, as primordial and preantral follicles—the populations most likely to respond to PRP—reside primarily in the cortex [48]. Other methodological concerns include the absence of a sample size or power calculation, which compromises the reliability of the findings. This is particularly important given that underpowered studies cannot provide statistically robust conclusions. In addition, embryos and pregnancy outcomes—arguably the most clinically relevant endpoints—were designated as secondary rather than primary outcomes, thereby diminishing the translational value of the results. The timing of ovarian stimulation following PRP administration also raises questions. The protocol allowed for up to four follow-ups over 3–6 weeks, meaning some patients initiated stimulation nearly three months post-PRP, by which time any biological effect may have waned. Since the interval between PRP and stimulation is critical to efficacy, such variability introduces heterogeneity and complicates interpretation. Finally, the ovarian stimulation strategy warrants scrutiny. The study employed high-dose gonadotropins (averaging 2000 IU) in women with poor ovarian response. While this approach is common in ART, emerging evidence suggests that excessive dosing may impair oocyte and embryo quality [49]. Indeed, the reported yield of only 2–3 oocytes could likely have been achieved with milder stimulation (e.g., clomiphene citrate protocols) without the potential drawbacks of supraphysiologic gonadotropin exposure.

Additionally, the trial by Barrenetxea et al. [9] had several methodological concerns, noting that (1) PRP was prepared and stored frozen rather than freshly processed, potentially impairing platelet function; (2) that injections were delivered into the ovarian medulla rather than the cortex where primordial follicles reside; and that (3) administration coincided with oocyte retrieval, a stage marked by luteinization and vascularization that may limit PRP’s effectiveness. The trial was also unclear regarding the number of injections per ovary, as well as questionable assumptions used in sample size calculations, particularly given the heterogeneity of three different ICSI cycles per patient. Despite these limitations, that trial is an important step, standardized protocols for PRP preparation, dosage, injection site, timing, and trial design are needed before definitive conclusions can be drawn, and until then, the utility of intraovarian PRP in DOR remains open for debate [25].

In PCOS, preclinical models indicate that PRP may restore endocrine balance, improve folliculogenesis, and mitigate oxidative stress and apoptosis, while isolated case reports in humans suggest resumption of ovulation and improved oocyte quality.

At the uterine level, intrauterine PRP infusion has been shown in multiple RCTs and cohort studies to enhance EMT, implantation, and clinical pregnancy rate in women with thin EMT or RIF, with meta-analyses confirming improvements in live birth rate. Nonetheless, not all studies are consistent, with some reporting no differences compared with controls, particularly in live birth outcomes. Importantly, hysteroscopic PRP injection into the subendometrial zone has recently emerged as a potentially more effective delivery method than intrauterine infusion, though these findings are based on small cohorts.

The heterogeneity of results underscores several critical challenges. First, PRP preparation techniques (single vs. double centrifugation, activation method, platelet concentration) vary widely, impacting growth factor content and biological activity. Second, injection timing (early follicular vs. peri-retrieval vs. luteal phase), site (cortical vs. medullary), and number of punctures differ across studies, complicating comparisons. Third, most available studies are small, non-randomized, and subject to selection bias. Fourth, outcomes often emphasize intermediate endpoints (hormonal markers, AFC, EMT) rather than live birth, which remains the most clinically relevant metric. Furthermore, while PRP is autologous and generally considered safe, concerns remain about procedural risks, potential for infection, and theoretical risks of abnormal angiogenesis or placentation.

## 5. Conclusions

PRP has emerged as a novel, autologous, and biologically plausible therapy for addressing some of the most challenging conditions in reproductive endocrinology, including DOR, POI, PCOS, thin endometrium, and RIF. Across ovarian applications, PRP has shown potential to improve surrogate markers of ovarian reserve, oocyte competence, and even embryo euploidy, while in the uterine setting, randomized and non-randomized studies suggest meaningful gains in EMT, receptivity, and pregnancy outcomes. Hysteroscopic PRP administration, though more invasive, may provide enhanced benefits compared to intrauterine infusion. Preclinical data further support the notion that PRP exerts multi-level effects through angiogenic stimulation, attenuation of oxidative stress, anti-apoptotic signaling, modulation of inflammation, and possibly epigenetic regulation of gamete competence.

Nevertheless, the current body of evidence remains limited by heterogeneity in PRP preparation protocols, variability in injection site and timing, small sample sizes, and a reliance on intermediate outcomes rather than definitive endpoints such as live birth. Concerns regarding safety, particularly abnormal placentation following intrauterine PRP, underscore the need for vigilance and long-term follow-up.

Future research should therefore prioritize well-designed, multicenter randomized controlled trials with standardized preparation and administration protocols, stratified by patient phenotype (DOR, POI, PCOS, thin EMT, RIF), and adequately powered to assess live birth rate as the primary endpoint. Comparative studies are also warranted to determine whether single versus repeated intraovarian PRP injections confer differential benefits, and whether hysteroscopic delivery provides superior efficacy over transcervical infusion. Mechanistic studies are equally important to elucidate the molecular pathways of PRP action, including its angiogenic, antioxidant, anti-apoptotic, and epigenetic effects.

Until such robust evidence is available, PRP should remain an experimental adjunct and be used cautiously in clinical practice. With further refinement and validation, PRP may ultimately evolve into a safe, reproducible, and transformative therapy in the management of infertility. We recommend that, in couples who are emotionally vulnerable after unsuccessful programs, treatments without well designed randomized trials be proposed with great caution. As such interventions are not part of established medical practice, charging high payments for them should be carefully reconsidered in the interest of fairness and professional integrity.

## Figures and Tables

**Table 1:** Summary of Human Studies on Intraovarian PRP for Ovarian Aging/Insufficiency.

Author/Year	Study Design/Population	PRP Method	Outcomes	Conclusions
Sills et al. [31]	4 women with DOR, poor IVF response	Autologous PRP + Ca gluconate, intraovarian	↓ FSH (13.6 → 7.7, *p* < 0.01); ↑ AMH (ns); 4–7 mature oocytes; 1 clinical pregnancy	PRP may improve ovarian reserve and IVF outcomes in DOR
Hosseinisadat et al. [32]	Before–after, 22 infertile women with DOR (Bologna criteria)	Autologous PRP after oocyte retrieval	↑ AMH (0.24 → 0.99, *p* < 0.001); ↑ AFC (ns); age/BMI no effect	PRP improved AMH, trend for AFC, independent of age/BMI
Yu et al. [33]	Prospective case–control, 74 IVF patients with ≥2 failed cycles	Autologous PRP bilateral ovarian cortex, 4 sites, follicular phase	↑ fertilized oocytes, blastocysts, and good-quality blastocysts (all *p* < 0.05); 29% pregnancy rate; benefits strongest 1–2 months post-PRP	PRP improved blastocyst yield/quality, short-term benefits
Merhi et al. [20]	Prospective, 12 infertile women with failed IVF	Autologous intraovarian PRP; IVF with PGT-A pre/post	Euploidy ↑ (8.1% → 39.3%, *p* = 0.002); 3 clinical pregnancies	PRP may enhance oocyte competence and embryo genetics
Potiris et al. [34]	Prospective cohort, 32 women ≥40 with anovulatory infertility	PRP from 65 to 70 mL blood, bilateral ovarian cortex, 2 courses	↑ AFC ( +75%); ↓ FSH, LH, prolactin; improved metabolic markers; clinical outcomes not reported	PRP improved ovarian/endocrine function, clinical impact unclear
Molinaro et al. [35]	Retrospective multicenter, 353 women ≤45 (207 DOR, 146 POI)	Autologous PRP + CaCl_2_, bilateral intraovarian	DOR: ↑ AFC, maturation, fertilization, cleavage; trend for ↑ pregnancy/live birth (7 live births)	PRP improved quality but not quantity; minimal effect in POI
Barrenetxea et al. [36]	RCT, 60 women with DOR (POSEIDON 3–4)	Autologous PRP 4 mL/ovary vs. saline; during oocyte retrieval	↑ mature oocytes (10.45 vs. 8.91, *p* = 0.008); no improvement in blastocysts, euploidy, or live births; higher pregnancy in controls (60% vs. 27%)	PRP increased oocyte yield but no benefit on genetics or outcomes; methodological issues noted
Li et al. [37]	Prospective, 71 women with poor ovarian response (POSEIDON 3–4)	Single vs. double intraovarian PRP injections	↑ AMH (0.33→0.43, *p* = 0.005), ↑ AFC (2.62→3.80, *p* < 0.001), ↑ retrieved oocytes and embryos; no difference between 1 vs. 2 injections	Single injection may be as effective as two, simpler and cost-effective

**Abbreviations:** DOR = Diminished Ovarian Reserve; POI = Primary Ovarian Insufficiency; PRP = Platelet-Rich Plasma; IVF = In Vitro Fertilization; AMH = Anti-Müllerian Hormone; FSH = Follicle-Stimulating Hormone; AFC = Antral Follicle Count; E2 = Estradiol; PGT-A = Preimplantation Genetic Testing for Aneuploidy; RCT = Randomized Controlled Trial; BMI = Body Mass Index; COH = Controlled Ovarian Hyperstimulation. ↑ means “increase in”, ↓ means “decrease in”, → means “from x value to y value”. ns: not significant.

## Data Availability

No new data were created.

## Abbreviations

AFC—Antral Follicle Count; AMH—Anti-Müllerian Hormone; ART—Assisted Reproductive Technology; COH—Controlled Ovarian Hyperstimulation; CRP—C-Reactive Protein; DHEA—Dehydroepiandrosterone; DOR—Diminished Ovarian Reserve; E2—Estradiol; EGF—Epidermal Growth Factor; EMT—Endometrial Thickness; ERα/ERβ—Estrogen Receptor alpha/beta; FET—Frozen Embryo Transfer; FGF—Fibroblast Growth Factor; FSH—Follicle-Stimulating Hormone; G-CSF—Granulocyte Colony-Stimulating Factor; GLUT4—Glucose Transporter Type 4; GnRH—Gonadotropin-Releasing Hormone; GSH-px—Glutathione Peroxidase; HCG—Human Chorionic Gonadotropin; HOXA10—Homeobox A10; HRT—Hormone Replacement Therapy; ICSI—Intracytoplasmic Sperm Injection; IGF—Insulin-like Growth Factor; IL-6/IL-10—Interleukin-6/Interleukin-10; IUI—Intrauterine Insemination; IVF—In Vitro Fertilization; LBR—Live Birth Rate; LH—Luteinizing Hormone; LIF—Leukemia Inhibitory Factor; MDA—Malondialdehyde; MII—Metaphase II (mature oocyte); MMPs—Matrix Metalloproteinases; NF-κB—Nuclear Factor Kappa-light-chain-enhancer of Activated B cells; PCOS—Polycystic Ovary Syndrome; PDGF—Platelet-Derived Growth Factor; PGT-A—Preimplantation Genetic Testing for Aneuploidy; PI3K/AKT—Phosphoinositide 3-Kinase/Protein Kinase B Pathway; P4—Progesterone; POI—Premature Ovarian Insufficiency; PRP—Platelet-Rich Plasma; RCT—Randomized Controlled Trial; RIF—Recurrent Implantation Failure; ROS—Reactive Oxygen Species; SOD—Superoxide Dismutase; SCF—Stem Cell Factor; T—Testosterone; TAC—Total Antioxidant Capacity; TGF-β—Transforming Growth Factor Beta; TNF-α—Tumor Necrosis Factor Alpha; VEGF—Vascular Endothelial Growth Factor.
